# Hierarchical modelling of crossing fibres in the white matter

**DOI:** 10.1162/imag_a_00436

**Published:** 2025-01-15

**Authors:** Hossein Rafipoor, Frederik J. Lange, Christoph Arthofer, Michiel Cottaar, Saad Jbabdi

**Affiliations:** Wellcome Centre for Integrative Neuroimaging, FMRIB, Nuffield Department of Clinical Neurosciences, Oxford, United Kingdom

**Keywords:** diffusion MRI, crossing fibres, fixel-based analysis, microstructural modelling, fibre-specific metrics, white matter fibre template

## Abstract

While diffusion MRI is typically used to estimate microstructural properties of tissue in volumetric elements (voxels), more specificity can be obtained by separately modelling the properties of individual fibre populations within a voxel. In the context of cross-subjects modelling, these fixel-based analyses are usually performed in two stages. Crossing fibre modelling is first performed in each subject to produce fixels, and these are subsequently modelled across subjects following registration and fibre population reassignment. Here, we introduce a new hierarchical framework for fitting crossing fibre models to diffusion MRI data in a population of subjects. This hierarchical setup guarantees that the crossing fibres are consistent by construction and, therefore, comparable across subjects. We propose an expectation-maximisation algorithm to fit the model, which can scale to large number of subjects. This approach produces a template for crossing fibre populations in the white matter which can be used to estimate fibre-specific parameters that are consistent across subjects, hence providing data that are by construction suitable for fixel-based statistical analyses.

## 1 Introduction

Diffusion MRI is widely used to study between-subjects variations in white matter, both in healthy cohorts and in patient populations (Johansen-Berg & Behrens, 2014;Jones, 2010). Like in voxel-based morphometry for studying grey matter (Ashburner & Friston, 2000), comparing white matter between subjects requires care in the between-subjects spatial alignment. This is particularly crucial when the objective of the analysis is the comparison of microstructural effects (from diffusion MRI models), rather than macroscopic variations in gross anatomy (S. M. Smith et al., 2006).

Recently, fixel-based analysis of white matter has emerged as a technique for cross-subject analysis of white matter not only within each voxel, but also within each fibre population in the voxel (Dhollander et al., 2021;D. A. Raffelt et al., 2017). In fixel-based analysis, a white matter orientation model is used to assign properties to fibre populations crossing within each voxel (e.g., the density of fibres aligned with a given direction). These metrics are then compared across subjects, allowing investigation of fibre-specific changes. This approach is in contrast to more traditional analyses that use voxel-wise metrics, such as fractional anisotropy (FA) or mean diffusivity (MD), as markers of white matter. While these voxel-wise metrics have been shown to be highly sensitive to changes in white matter tissue (e.g.,Scholz et al., 2009), they cannot easily be interpreted in terms of specific changes to the underlying fibre populations within the voxel (seeFig. 1).

**Fig. 1.**
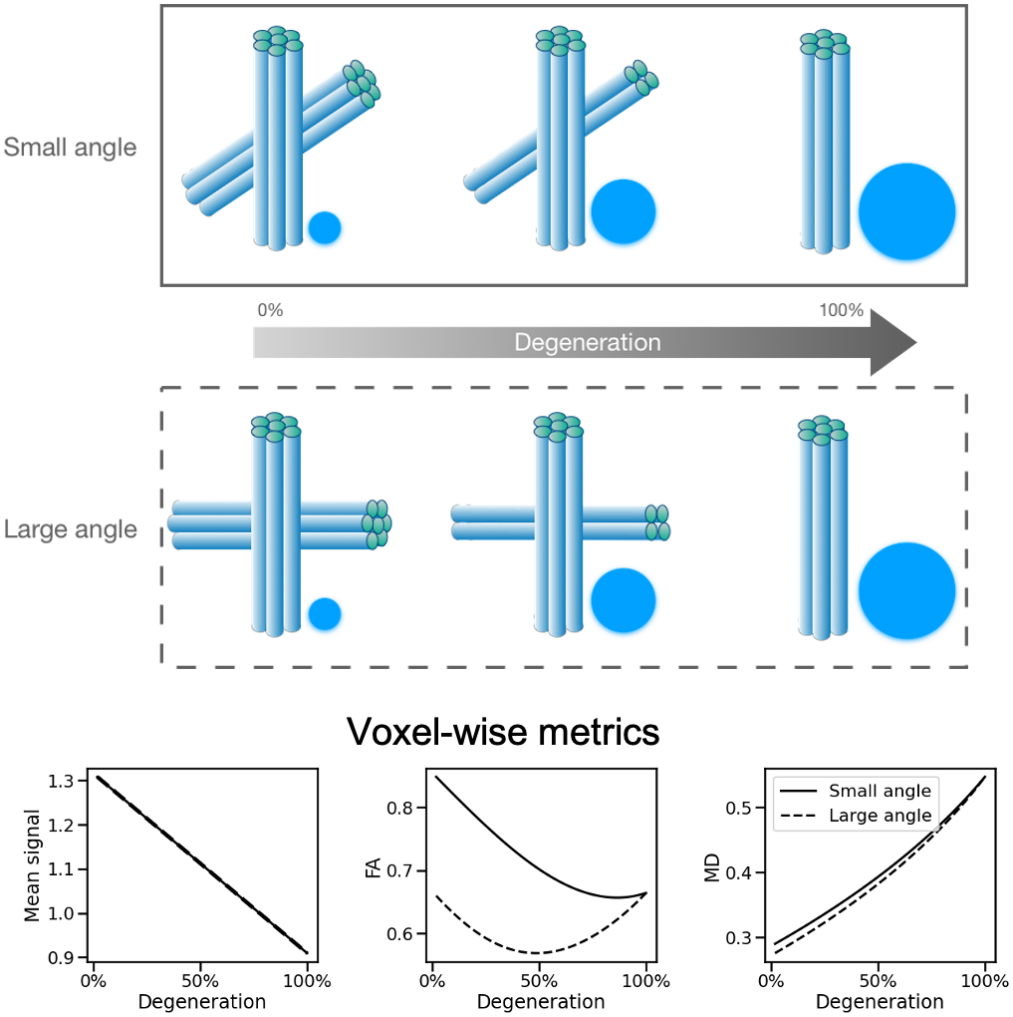
Illustration of the effect of a fibre-specific biological process on voxel-wise measurements. The top panels show a scenario of axonal degeneration in a crossing fibre region, where one of the fibre populations (shown by cylinders) gradually degenerates and is replaced by water with isotropic diffusion (shown by circles). Two possible scenarios for the same process are depicted in the two panels, one in which the angle between the two fibre populations is small, and another in which the angle is large. The bottom row shows the change in voxel-wise measurements of mean signal, fractional anisotropy, and mean diffusivity in both scenarios (solid line for the small angle and dashed line for the large angle). The angle between the fibres has no effect on the mean signal and a small effect on MD, but it substantially affects the changes seen in FA.

Fixel-based approaches attempt to resolve this fibre-specific issue. By assigning measures to a discrete set of fibre orientations within a voxel, between-subjects differences can then be associated with specific fibre orientations, which in turn may correspond to fibre pathways associated with specific functions. However, not only do fixel-based analyses need to resolve between-subject spatial alignment (just like voxel-wise methods do), they also need to resolve between-subject assignment of fibres. In order to “compare like with like”, the discrete set of fibres within a voxel must all be in correspondence across subjects (Jbabdi et al., 2010).

So far, this between-subject fibre population assignment has been conducted as a post-processing step, following independent processing of each subject separately. InJbabdi et al. (2010), fibre orientations are estimated on a subject-by-subject basis using the ball-and-sticks model (Behrens et al., 2007). Then, to maximise correspondence both spatially and between subjects, fibre labels are swapped in multiple stages. The method was restricted to the white matter skeleton (S. M. Smith et al., 2006) in order to ensure validity of the spatial assignment. More recently, fixel-based analyses were conducted using continuous fibre Orientation Distribution Function (fODF) models (D. A. Raffelt et al., 2017;Tournier et al., 2019) fitted to each subject separately. Peaks from the fODF were subsequently matched and relabelled across subjects. This approach is, in fact, what is currently referred to as fixel-based analysis.

However, even after accurately reorienting fibre populations through spatial normalisation, assigning fibres across subjects post-hoc can occasionally be problematic. One-to-one mapping between each subject’s peaks and the template peaks can sometimes fail when multiple peaks in a subject’s ODF correspond to the same fibre population (due to non-ideal parameter estimation or the presence of fibre dispersion modelled as narrow fibre crossing) or when a weak fibre population fails to be reconstructed in a subject as a separate peak due to noise.

In this paper, we propose a solution to this problem that takes part earlier in the processing pipeline, at the stage of voxel-wise white matter orientation modelling, rendering post-hoc fibre reassignment unnecessary. We build a cross-subject hierarchical model of crossing fibres where, in each voxel, the parameters of the model (including fibre orientations) are drawn from a population distribution. This means that the assignment of the fibre orientations is matched across subjects by construction, rather than through a post-processing step. We fit both the individual subjects and the group parameters simultaneously to all the data and obtain group and individual estimates of orientations and associated fixel parameters. After a template has been created, it can be used to compare signal fractions in different fibres across a larger sample of individuals by fitting the ball-and-sticks model with the template acting as the prior distribution.

In addition, we propose an expectation-maximisation algorithm that enables us to fit the model in a way that is scalable to a large cohort of subjects. We also extend the voxel-based Threshold-Free Cluster Enhancement (TFCE) approach (S. Smith & Nichols, 2009) to enable statistical analysis of fixels with proper correction for multiple comparisons across space. The method and implementation are validated through simulations. Finally, we create a group-averaged fixel template for white matter using data from the UK Biobank (Miller et al., 2016), and we used this template in an example analysis of fixel changes with ageing.

## 2 Theory

### 2.1 Crossing fibre model

While our framework can be adapted to work on different crossing fibre models, we choose the ball-and-sticks model because it is simple, widely used, and it lends itself naturally to a hierarchical extension. According to this model, the diffusion signal in a voxel originates from the combination of a ball compartment that represents water with isotropic diffusion and one or more stick compartments that represent water that diffuses along a fibre population. The diffusion signal according to the ball-and-sticks model is generated by

$$S=S_{0}\left[\sum_{i}f_{i}e^{-bd{\left(\mu_{i}^{T}g\right)}^{2}}+(1-\sum_{i}f_{i})e^{-bd}\right],$$ (1)

where b, g are the b-value and the gradient orientation, d is the diffusion coefficient,$\mu_{i},f_{i}$are the orientation and signal fraction of the i’th stick, and$S_{0}$is the baseline signal with no diffusion encoding. Hence, the set of free parameters in this model is$\left\{S_{0},d,f_{i},\mu_{i}\right\}$per subject per voxel.

### 2.2 Hierarchical model

In a hierarchical framework, we assume that, in each voxel, each subject’s parameter ($\nu_{s}$) represents a sample drawn from a population distribution. This hierarchical structure ensures that all parameters, including the labelling of the fibre populations, are consistent across subjects.

The population distributions of all scalar parameters are assumed to be Gaussian with unknown means and variances, while orientation parameters are assumed to follow a Watson distribution with unknown mean and concentration. Finally, each subject’s diffusion MRI signal is derived from the subject parameters following the forward model (M) inEquation 1with additive noise ($D_{s}=M\left(\nu_{s}\right)+\in$). The noise distribution is assumed to be a zero-centred Gaussian with a variance ($\sigma_{s}^{2}$). This variance also has an exponential prior distribution with hyperparameter$\alpha_{n}$. The diagram inFigure 2shows the full generative forward model. Using the dependency structure that is shown in the graph, the joint distribution of all free parameters factors into:

**Fig. 2.**
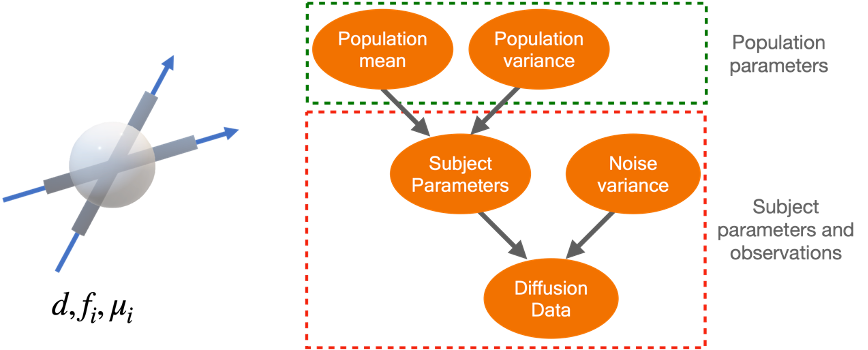
The generative forward model of diffusion MRI signals for the whole population. Left: A schematic representation of the ball-and-sticks model for diffusion MRI signal in white matter voxels. According to this model, water only diffuses axially inside each population of fibres and isotropically outside the fibres. Right: The hierarchical graphical model for the diffusion MRI signal in a white matter voxel for a population of subjects. In this model, we assume that, within a single subject, each parameter of the ball-and-stick model is drawn from a population distribution. The red box shows subject-specific parameters and observations, and the green box shows population-level parameters. This hierarchical structure will introduce a dependency between the subjects, which has the advantage of ensuring that the fibre labels are consistent across subjects, therefore, allowing for the comparison of fibre-specific parameters between subjects.

$$\begin{array}{l} P\left(\left\{\nu_{s}\right\},\left\{\sigma_{s}^{2}\right\},\nu_{g},\sigma_{g}^{2},\left\{D_{s}\right\}\right)\\ \;\;\;=\left(\prod_{s}P\left(D_{s}\text{|}\nu_{s},\sigma_{s}^{2}\right)P\left(\sigma_{s}^{2}\right)P\left(\nu_{s}\text{|}\nu_{g},\sigma_{g}^{2}\right)\right)\times P\left(\nu_{g}\right)P\left(\sigma_{g}^{2}\right). \end{array}$$ (2)

The curly brackets indicate sets of subject-specific parameters. The conditional probabilities in the graphical model are defined as

$$\begin{array}{cccc} P(D_{s}|\nu_{s},\sigma_{s}^{2}) & = & Normal\left(M\left(\nu_{s}\right),\sigma_{s}^{2}I\right) & \\ P(\nu_{s}|\nu_{g},\sigma_{g}^{2}) & = & Normal\left(\nu_{g},\sigma_{g}^{2}\right) & \text{for scalar}\nu_{s}\\ P(\nu_{s}|\nu_{g},\sigma_{g}^{2}) & = & Watson\left(\nu_{g},\sigma_{g}^{2}\right) & \text{for directional}\nu_{s}. \end{array}$$ (3)

For the Watson distribution,νis the mean orientation, and$\sigma_{g}^{2}$is the orientation dispersion index (H. Zhang et al., 2012). We assume uniform priors within plausible ranges for all the group mean parameters ($\nu_{g}$) and a power-law prior for the group variances ($\sigma_{g}^{2}$) with hyperparameter$\alpha_{g}$. The power-law prior is used to prevent the group variance from going to zero. The prior distributions of the parameters are given by

$$\begin{array}{ccc} P\left(\nu_{g}\right) & \propto & 1\\ P\left(\sigma_{g}^{2}\right) & \propto & e^{\alpha_{g}\sigma_{g}^{2}}\\ P\left(\sigma_{s}^{2}\right) & \propto & e^{-\alpha_{n}\sigma_{n}^{2},} \end{array}$$ (4)

where$\alpha_{g}$and$\alpha_{n}$(positive real numbers) are hyperparameters that are adjusted by the user.

### 2.3 Parameter inference

We employ a maximum a-posteriori approach to fit the parameters of the hierarchical model to the data, that is to maximise the conditional probability of all the parameters of interest given the data ($P\left(\nu_{g},\sigma_{g}^{2},\left\{\nu_{s}\right\},\left\{\sigma_{s}^{2}\right\}|\left\{D_{s}\right\}\right)$). The free parameters of this model are the group means and variances for each model parameter, subject parameters, and subject-specific noise variance. The full posterior probability of the model according to the hierarchical structure factorises into

$$\begin{array}{l} P\left(\left\{\nu_{s}\right\},\left\{\sigma_{s}^{2}\right\},\nu_{g},\sigma_{g}^{2}\text{|}\left\{D_{s}\right\}\right)\\ \;\;\;\propto \left[\prod_{s}P\left(D_{s}\text{|}M\left(\nu_{s}\right),\sigma_{s}^{2}\right)P\left(\nu_{s}\text{|}\nu_{g},\sigma_{g}^{2}\right)\right]\times P\left(\nu_{g}\right)P\left(\sigma_{g}^{2}\right). \end{array}$$ (5)

Fitting this entire model requires simultaneously optimising all of these parameters, which creates a space with a very high number of dimensions that grows linearly with the number of subjects. This makes optimisation infeasible for datasets with more than a few tens of subjects.

To make the optimisation feasible for large datasets, we employ an expectation-maximisation approach: In the expectation step, the group parameters are estimated while the subject parameters are fixed; in the maximisation step, the group parameters are held fixed while the subject parameters are estimated. These stages are repeated until all of the parameters have converged. Convergence is achieved when all group and subject parameters remain unchanged between subsequent iterations.

### 2.4 Expectation

In the expectation step, the subject parameters are set to the best guess made so far so that the population parameters can be estimated. FollowingEquation 5, this leads to optimising the following conditional posterior:

$$\begin{array}{l} P\left(\nu_{g},\sigma_{g}^{2}\text{|}\left\{\nu_{s}\right\},\left\{\sigma_{s}^{2}\right\},\left\{D_{s}\right\}\right)\\ \;\;\;\propto \prod_{s}P\left(\nu_{s}\text{|}\nu_{g},\sigma_{g}^{2}\right)\times P\left(\nu_{g}\right)P\left(\sigma_{g}^{2}\right). \end{array}$$ (6)

Since we assume that there is no correlation between parameters a-priori, each parameter is evaluated on its own at this stage. For the scalar parameters, plugging the probabilities according toEquation 6results in

$$\nu_{g}=\frac{1}{n}\sum_{\text{s}}\nu_{s},\sigma_{g}^{2}=\frac{\frac{1}{2}\sum_{s}{\left(\nu_{g}-\nu_{s}\right)}^{2}}{\alpha_{g}+1+\frac{n}{2}}.$$ (7)

Numerical optimisation is used for the maximisation of the posterior to estimate group orientation parameters, including average orientation and dispersion for each fibre population. For all the model fittings (both for individual fits and in the hierarchical model), we have used non-linear model fitting as implemented in the SciPy package Nelder–Mead method (Nelder & Mead, 1965;Virtanen et al., 2020).

### 2.5 Maximisation

In this stage, the population mean and variance parameters are fixed, and the subject parameters are estimated by maximising the conditional posterior probability. According toEquation 5:

$$\begin{array}{l} P\left(\left\{\nu_{s}\right\}|\nu_{g},\sigma_{g}^{2},\left\{D_{s}\right\}\right)\\ \;\;\;=\prod_{s}P\left(D_{s}\text{|}\nu_{s},\sigma_{s}^{2}\right)P\left(\nu_{s}\text{|}\nu_{g},\sigma_{g}^{2}\right)P\left(\sigma_{s}^{2}\right). \end{array}$$ (8)

Since the group parameters are fixed in this stage, the likelihood is independent for each subject and factorises into subject-specific parts. This makes it possible to optimise each subject’s parameters separately. We, therefore, independently estimate subject parameters$\nu_{s}$by optimising the logarithm of the posterior function through numerical optimisation.

### 2.6 Determining the number of fibres in each voxel

To determine the number of fibre populations in each voxel, we first fit the hierarchical model using one, two, and three fibres. We then set a threshold on the difference in the quality of the fit achieved by adding a fibre population. The log-likelihood of the data$\text{log}(P(D_{s}|\nu_{s},\sigma_{s}^{2}))$summed over all subjects is used as the quality of fit metric.

**Fig. 3.**
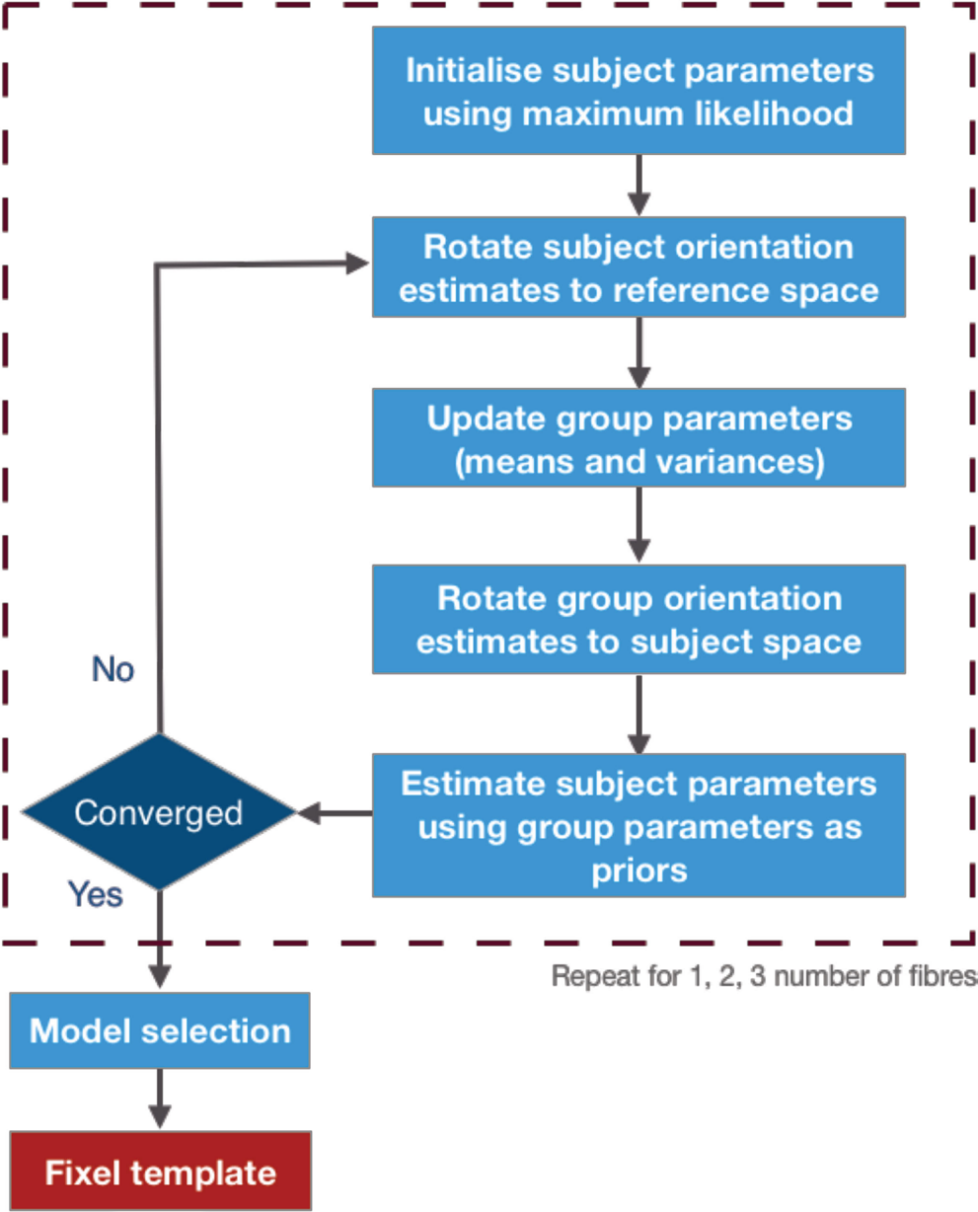
Template creation pipeline. Firstly, the diffusion data are fitted with models for each subject independently using maximum likelihood estimation. We then use the parameters from all subjects to estimate the population distribution for each parameter. We then rotate the orientations back into the space of each subject. The population parameters (mean and variance) are then utilised as prior distributions to re-fit the subject parameter to the diffusion data. This process is repeated until convergence, and for models with one, two, and three fibres separately. In order to determine the number of fixels within a voxel, we first estimate the improvement in the likelihood function from one to two fibres, and from two to three fibres. Then, we apply thresholds to these improvements to identify the number of fixels in each voxel. This gives us a fixel template that we can use as a prior distribution to fit fixels to each subject. The inputs of the pipeline are diffusion data for each subject that is resampled into a structural template space, and transformations from each subject’s diffusion data to that template. Note that all the computations happen for each voxel in the reference space and all the diffusion data are only fetched once needed, so there is no need to register the diffusion data into the template space. To accomplish this, for every voxel in the reference space, we assign a matching voxel from each subject, that is the closest voxel in the subject’s space after transforming the coordinates.

Figure 3shows the full pipeline for fitting the hierarchical model, including the creation of a white matter fixel template representing the group average orientations and population volume fractions in the model.

### 2.7 Fixel-based threshold-free cluster enhancement

The natural next step following fitting fixels is to fit a cross-subject model of the parameters, for example, the fibre signal fractions, using a general linear model (GLM). As this is done in each voxel, statistical techniques for controlling false positives are required due to the large number of tests involved. One popular technique is Threshold-Free Cluster Enhancement (TFCE) (S. Smith & Nichols, 2009), which is a method for multiple comparison correction developed for voxel-wise cross-subject analyses. TFCE works in an analogous way to cluster-based thresholding methods, but without requiring a cluster-forming threshold. Here, we show how we can extend TFCE to the context of fixel-based analyses.

TFCE works by forming contiguous clusters of voxels that are above a given statistical threshold, then calculating a statistic that combines the cluster height and extent, and finally integrating this statistic across different thresholds. To apply this method to fixel data, we need to modify the definition of spatial neighbourhood used for forming clusters. An example of such modification is found inD. A. Raffelt et al. (2017), where they utilise tractography to establish the neighbourhood structure. In contrast, our approach employs a different rationale for defining the fixel neighbourhood.

In our definition, two fixels are regarded as neighbours if they are located in adjacent voxels (27-neighbourhood) and if the angle between them is below a predefined threshold (seeFig. 4). This notion of adjacency has three advantages: it is symmetric; it maintains adjacency between fixels that are potentially in the same tract; and, finally, it prevents information from being mixed between crossing fibres within the same voxel. We construct a graph of connections between fixels, where each node in the graph is a fixel, and two fixels that meet the above criteria for being neighbours share an edge.

**Fig. 4.**
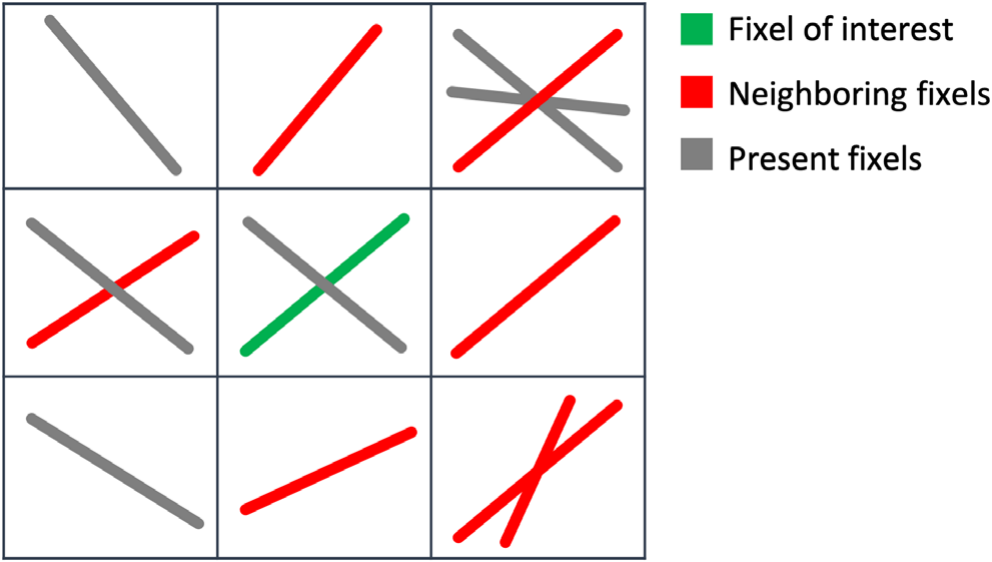
2D section of fixel neighbourhood for TFCE. The neighbours of a fixel (green fixel) are all the fixels that are located in an adjacent voxel (at least one corner is shared, in total 27 voxels) and make angles smaller than a threshold (red fixels). Multiple fixels in a neighbouring voxel may be taken into account as neighbours of the same fixel.

For the fixel of interest, the cluster extente(h)at threshold h is defined as the number of nodes in the connected component containing the fixel, only considering the sub-graph that includes fixels with statistics greater than h. As a result, we assign a TFCE score to each fixel as

$$TFCE=\int_{0}^{t}e{\left(h\right)}^{E}h^{H}dh.$$ (9)

Here E and H are parameters that weigh the importance of cluster extent and height, respectively,tis the statistic for the fixel of interest.

Since the TFCE scores may not have a Gaussian distribution, we use standard non-parametric permutation testing to estimate p-values for each fixel. In order to achieve this, we permute the original data labelling (for example, subjects between two groups for examining group differences) and re-estimate the TFCE score multiple times to produce a null distribution. The p-value is then calculated as the likelihood of observing the actual TFCE score or higher in that null distribution.

## 3 Methods

### 3.1 Simulation settings

To determine the effectiveness of the hierarchical framework, we evaluated it with simulated data. Diffusion data for 30 participants in a single crossing fibre voxel were simulated using a ball-and-sticks model with two fibres. Each subject’s parameters are randomly sampled from a population distribution. The signal fractions are sampled from Gaussian distributions with means0.5,0.3and standard deviation of0.1,0.1and then truncated to range[0,1]and normalised to sum to0.8. Therefore, on average the first fibre population is stronger, but that may not be the case for every single subject. The stick orientations are samples from a Watson distribution with low dispersion (ODI=0.1), so that each fibre population forms a cluster across subjects. Following that, using an acquisition protocol that includes 5 b0 images and 50 diffusion-encoded images withb=1,2ms/μm^2^(identical to that used by the UK biobank dataset (Miller et al., 2016), diffusion data for each subject are simulated using a custom python script. All diffusion signals are then distorted by adding Gaussian noise with SNR=50.

### 3.2 Data sampling and voxel correspondence

For the hierarchical framework to work, there needs to be spatial correspondence between subjects. This is achieved through image registration of each subject’s data to a common reference space. Registration was performed using FSL MMORF (Lange et al., 2020), a multimodal registration tool that uses both scalar structural (T1-weighted) images and diffusion tensor images (DTIs) to drive the registration. We used the Oxford-MultiModal-1 (OMM-1) template (Arthofer et al., 2021) as our standard reference space for voxel correspondence. OMM-1 is preferred over MNI152 since it was constructed using simultaneous alignment of T1-weighted, T2-FLAIR, and DTI images and, therefore, includes high-quality, unbiased, scalar, and tensor data in a common space.

It is noteworthy that MMORF is not the exclusive choice for generating warp fields for registering diffusion data to the template space. Nevertheless, the incorporation of orientation information within the registration procedure is essential. This incorporation guarantees the consideration of white matter orientation alignment during warp field creation. Alternative registration methodologies that integrate orientations into the alignment process, as demonstrated byD. Raffelt et al. (2011),H. Zhang et al. (2006), andP. Zhang et al. (2014), offer credible alternatives.

We chose not to resample the diffusion data into a shared reference space after registration due to memory and space constraints. Instead, we built correspondence by extracting the necessary data at each stage of inference using the warps between individuals and a reference space, with nearest neighbour interpolation. To move across individual and common spaces, we are required to map the voxel position as well as the registration-induced local re-orientation and apply them to the orientation parameters. Because registration was driven by directional information within the white matter, this step also contributes to reducing cross-subject variability in fibre orientation estimates. In order to rotate all orientation parameters, we used the warp field’s local Jacobian matrix. We chose not to isolate the rotation component from the Jacobian in order to also account for non-rotational types of distortion, such as shearing. Instead, we used the complete Jacobian matrix to transform the orientation vector and then re-normalised the resized orientations to unit length. For example, any given direction parameter in subject native space is resampled to the reference space using

$$\mu_{ref}=\frac{J\mu_{nat}}{\left|J\mu_{nat}\right|},$$ (10)

where$\mu_{nat}$is the orientation parameter in subject native space, J is the Jacobian matrix of the warp field at that voxel, and$\mu_{ref}$is the orientation after resampling to the reference space. A very similar approach has been employed in other studies, as demonstrated inAlexander et al. (2001),Hong et al. (2009), andD. Raffelt et al. (2011,^2012^).

### 3.3 Fixel template creation

To create a fixel template, we used the diffusion data of 100 unrelated subjects from the HCP dataset for young adults (Van Essen et al., 2013). The diffusion data include 3 shells (b-values:1,2,3ms/μm^2^with 90 orientations each) and 18 b0 images (in total 288 volumes). These data have been preprocessed using the standard preprocessing pipeline in FSL for the HCP dataset (Sotiropoulos et al., 2013).

Each subject was registered to OMM-1 space using MMORF. Registration was driven using brain-extracted T1-weighted and DTI modalities. As fixel analysis is only valid within white matter, we created a white matter mask by running FSL FAST (Jenkinson et al., 2012;Y. Zhang et al., 2001) on the T1-weighted image of the OMM-1 template. The template creation process then proceeded as described above. Following the creation of this template, its parameters were fixed and it was used as a prior distribution in order to fit the diffusion model to new, unseen subjects as detailed in the following section.

### 3.4 GLM analysis

In order to illustrate an example fixel-based analysis utilising the fixel template, we ran a GLM to model changes in fibre signal fractions with age. The diffusion data from 400 randomly selected subjects (200 male, age 50 +/- 10) in the UK biobank were used for the GLM analysis. This dataset contains 2 shells (b-values=1,2ms/μm^2^each in 50 directions) and 5 b0 images (105 volumes in total). The standard preprocessing pipeline for UK biobank data was used to preprocess the diffusion data (Alfaro-Almagro et al., 2018). Once again, MMORF registration was used to estimate warps for transforming parameters between individual’s diffusion space and the OMM-1 template space. In this instance, T2-FLAIR data were used in addition to T1-weighted and DTI data to drive the registration.

We used fixel signal fractions as dependent variables, and age as the independent variable in our GLM analysis, with sex and head size as confounding factors. The t-statistics for the regression against age were estimated using a linear fit for each fixel independently. Then, we estimated voxel-wise p-values from permutation tests (5000 permutations) using the fixel-based TFCE scores calculated with the proposed cluster enhancement method.

## 4 Results

### 4.1 Simulations

The individual (independent) fitting approach (maximum likelihood estimates) and hierarchical model are employed to estimate individual subject parameters and population-level distribution parameters from simulated diffusion data with known ground truth. The estimated parameters from each approach versus the actual parameters for each subject are shown inFigure 5.

**Fig. 5.**
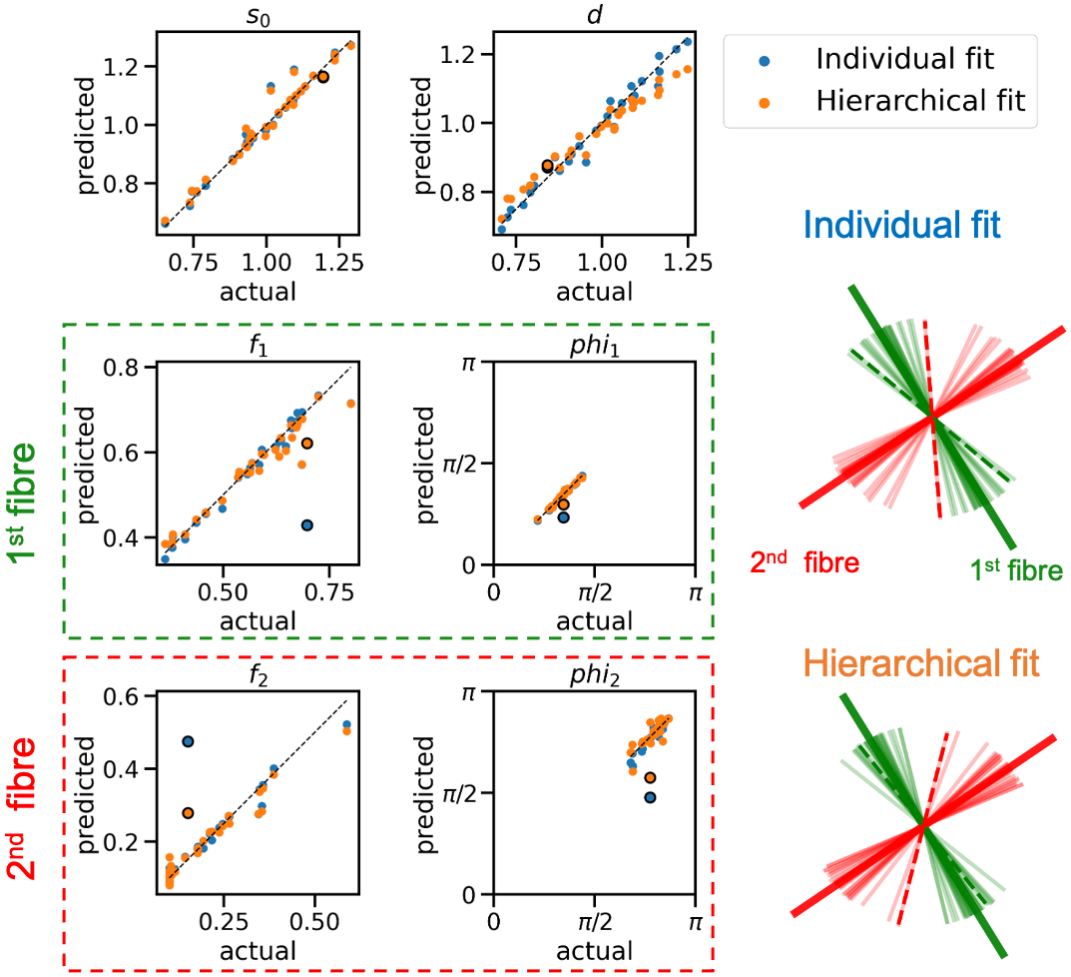
Scatter plots show estimated vs. actual parameters for the 2-fibre ball-and-stick model in a single voxel for 30 subjects. To facilitate visualisation of the orientation parameters, we fixed the spherical polar angle to 90 degrees for all subjects, displaying only the azimuthal angle phi. Each dot represents a subject, and proximity to the identity line indicates better parameter estimation accuracy. In individual fits, fibre populations are relabelled using a peak matching algorithm that iteratively minimises the sum of the distances to cluster centres and then updates the centres using new labels. For hierarchical fits, fibre populations are labelled based on the strength of the group-averaged fibre population. The right side of the figure shows the estimated orientations of the sticks for both individual and hierarchical fits as well as the true group average. For most subjects, the individual fit accurately estimated fibre population strength and orientations and correct labelling. However, for an exemplary subject (depicted with dashed lines), the two estimated fibre populations are close to one of the fibre clusters, making post-hoc relabelling ambiguous. The scatter plot of fibre population signal fractions reveals that for this subject (marked with a black circle), the signal fraction estimates are significantly biased due to inaccurate parameter estimation. The hierarchical fit for the same subject shows slightly different angles that are closer to the true value but substantially lower bias in the fraction estimates. This highlights the robustness of the hierarchical approach in maintaining accurate signal fraction estimates. Also, in this case, the estimates for the diffusivity show a slight bias at the higher end, likely due to the group prior pushing estimates towards the mean. Therefore, we recommend applying the priors only to angles and not to other parameters.

The results indicate that the hierarchical fit is more accurate for estimating the model parameters. In particular, the individual fitting approach failed to detect the weaker secondary fibre population in the majority of subjects (most of the second fibre signal fractions are zeros).

### 4.2 White matter fixel template

The white matter fixel template contains the mean and variance of the ball-and-sticks model parameters for each white matter voxel, including the diffusivity, direction, and signal fraction of the fibre populations in that voxel. This provides a basis for comparing the signal fractions in different fibre populations over a broader sample of participants by fitting the ball-and-sticks model using the template as prior.

**Fig. 6.**
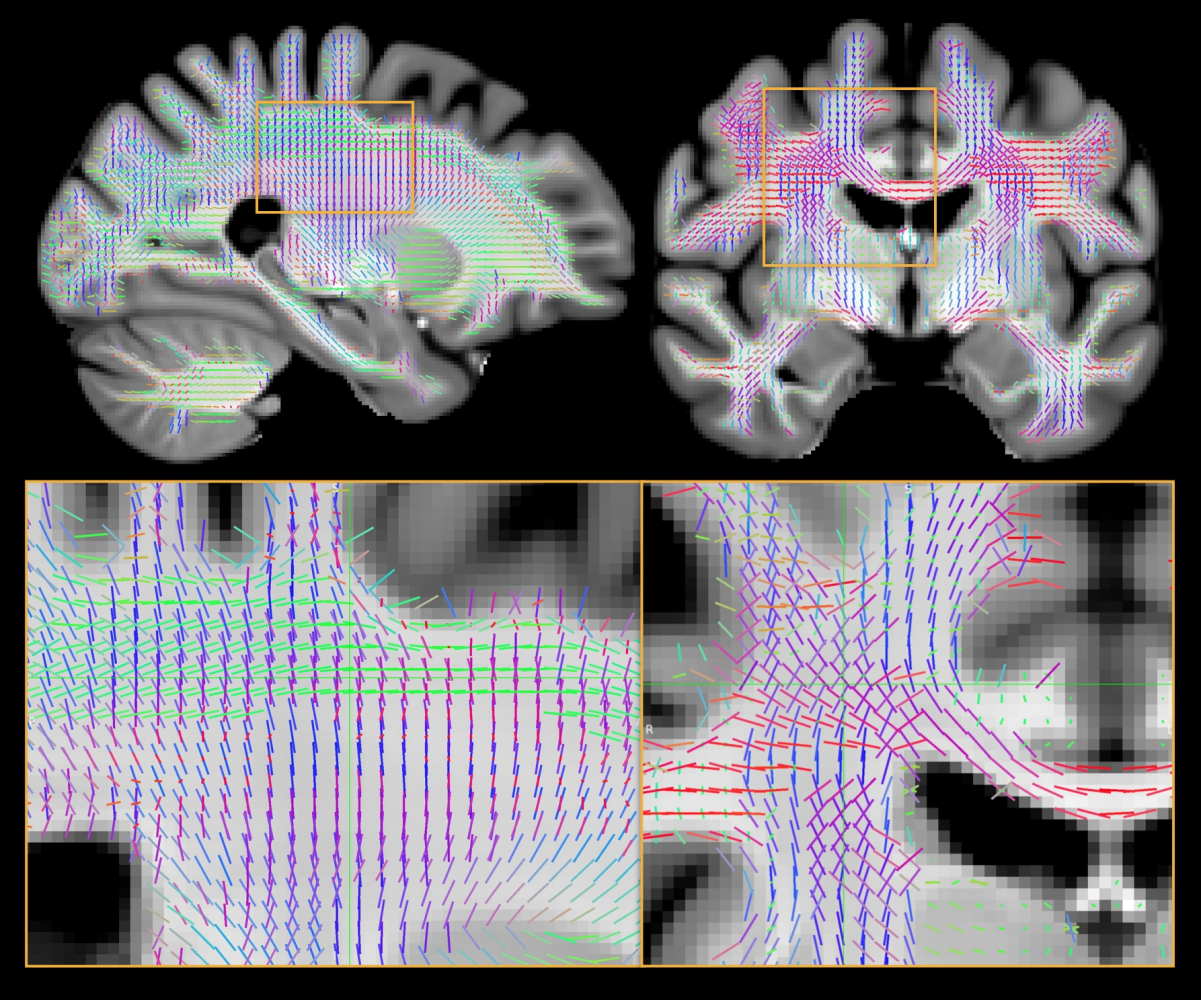
A sagittal and coronal slice of the white matter fixel template created using HCP diffusion data overlaid on the OMM-1 T1-weighted image. The fixels that are present in each voxel are depicted by lines that are colour coded according to their orientation (L-R: red, I-S: blue, A-P: green). The number of fixels is decided by thresholding the likelihood function improvement that is achieved by adding more sticks. Two sample patches of crossing fibre areas are magnified for better assessment. Although the technique does not involve regularisation across voxels, the fixels exhibit a high degree of spatial continuity. At the boundaries, we observe some fixels entering the grey matter radially.

Figure 6displays two sections of the template’s fixel orientations and two magnified areas of crossing fibre regions for better visual inspection. These orientations are calculated using the group average across subjects. Although there are no between-voxel spatial constraints, the orientations of the fixels exhibit good spatial continuity and conform to well-established anatomical features.

**Fig. 7.**
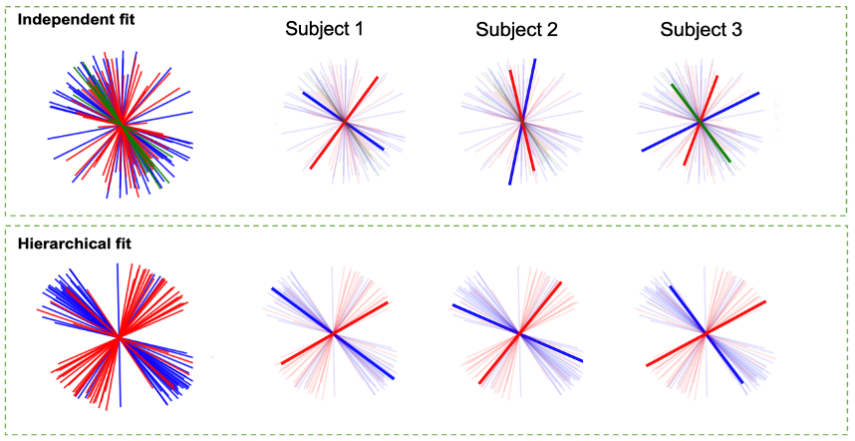
Estimated fibre orientations in a sample voxel in a crossing fibres region for 30 subjects from the UK Biobank data. The top panel shows the aggregation of all subject orientations, along with three representative subjects for individual model fitting followed by relabelling. The first, second, and third fibre populations are represented by blue, red, and green, respectively. Various types of variability are observed across subjects: In this voxel, Subject 1 shows a large angle between fibre populations, with the first and second fibre populations reversed compared with other subjects. In Subject 2, the fibre populations are oriented at an acute angle to one another, which suggests that they might correspond to the same group fibre population, and Subject 3 exhibits three distinct fibre populations. These differences along with the high dispersion in fibre orientations make post-hoc analysis to match fibre population labels between subjects challenging. The lower panel displays the hierarchical model parameters for the same data, with labelling based on the strength of the group average. In the aggregate plot, we observe that the fibres have organised into two distinct clusters. Similarly, the sample subjects demonstrate a strong correspondence with the template and consistency between them.

Figure 7compares the estimated fibre orientations for a sample white matter crossing fibre voxel with and without the hierarchical model. As a result of the subject-specific nature of the individual fit, existing fibre populations may not be oriented consistently across subjects, and some individuals may have a different number of fixels. Also, despite the fact that there is a high degree of consistency in the orientation of the fibre population between subjects, in some of them the relative strength of the fibre populations is swapped.

The same fit is displayed in the lower panel, but this time the hierarchical model with two fibre populations was used. The results demonstrate that the fibres are aligned in two distinct groups and are matched between subjects.

Maps and a histogram of the cross-subject variation in orientation of the first fibre population (all white matter voxels) are shown inFigure 8for both individual and hierarchical fits. The across subject’s variability of the major fibre orientation in the individual fit is substantially higher than that for the hierarchical fit. This is due, in part, to the fact that certain subjects’ first and second (or third) fibre populations have switched places, and also because the hierarchical fit tends to pull orientations closer to the group average when data are not strongly supportive of a particular orientation (e.g., in outlier or noisy subjects). The maps depict a slice of the cross-subject dispersion estimated in the white matter voxels. In the hierarchical fit, any high variance resides close to the grey matter boundaries, where there is no strong and consistent fibre orientation structure.

**Fig. 8.**
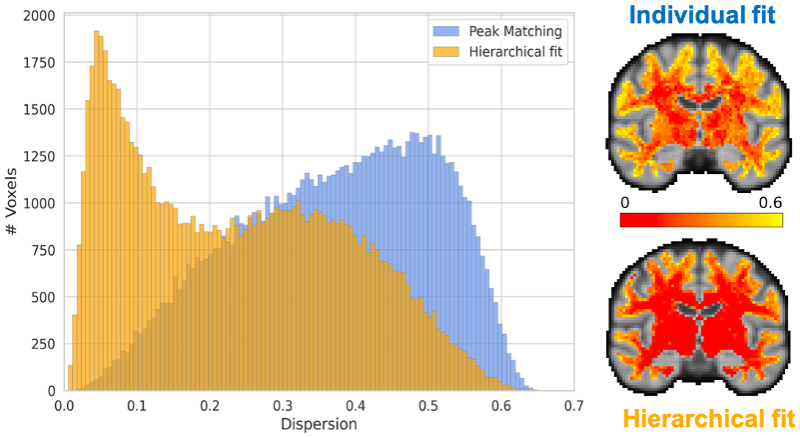
The histograms show the distribution of across subject dispersion in all white matter voxels for individual fits followed by peak matching and for the hierarchical fit. The dispersion along the x-axis is quantified using the orientation dispersion index, as defined inH. Zhang et al. (2012). This index is computed by fitting a Watson distribution to the subject orientations, estimating the concentration parameter, and subsequently converting it to the orientation dispersion index. A noticeable reduction in cross-subject variability is observed, indicating a higher degree of alignment among the primary fibre population across subjects for the majority of voxels. This reduction can be attributed to both the hierarchical framework drawing subject parameters towards the group average and also better labelling of fibre populations. The accompanying maps display dispersions for a single slice of the standard brain. In the hierarchical fit, it is evident that the majority of high-dispersion voxels are located at the grey matter boundaries, while fibre populations in the deep white matter exhibit increased alignment across subjects. In contrast, this pattern is not observed in the individual fitting approach, especially at the crossing fibre regions.

The number of fibre populations in each white matter voxel is depicted inFigure 9in example slices. The improvement in the likelihood function by adding an extra fibre population is the criterion for selecting the number of fibre populations in each voxel. The left histogram depicts the distribution of the difference in log-likelihood between models with two and one fibre population, summed across all participants. We apply a threshold to this histogram in order to identify voxels containing a single fibre population. The threshold is chosen by visually inspecting the maps to ensure a certain degree of symmetry across hemispheres and reasonable spatial homogeneity. The histograms on the top right display the distribution of the log-likelihood difference between the two and three fibre models, but only for the voxels that are over the one fibre threshold. We apply a threshold to this histogram in order to identify voxels with two or three fibre populations.

**Fig. 9.**
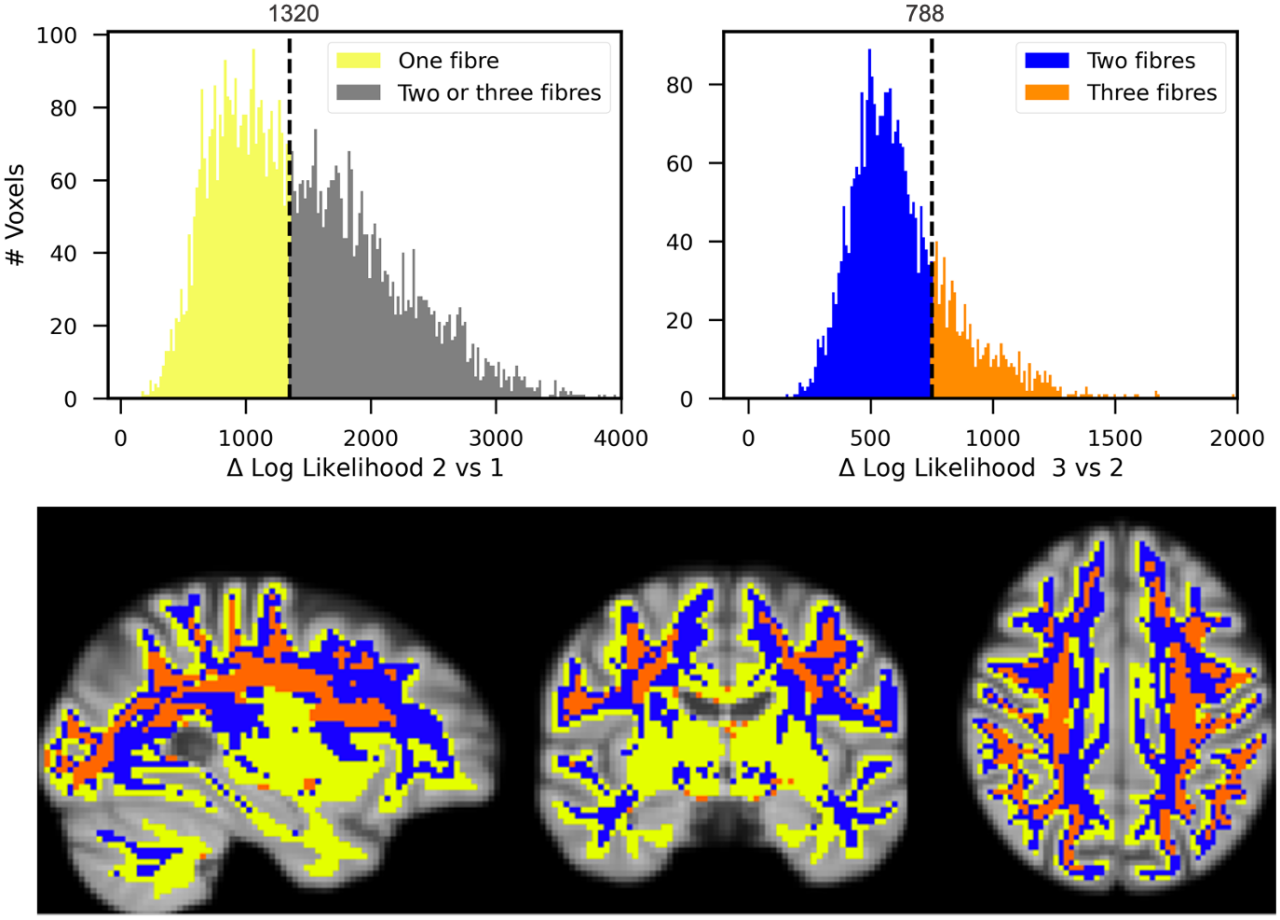
Determining the number of fixels present in each voxel. The histogram on the left displays the distribution (across the voxels in the white matter mask) of the increase in subject-averaged log-likelihood from one to two fibre models. We expect a better fit to data with more free parameters; hence all numbers are positive. To determine which voxels contain only one fibre population, we threshold these differences. The threshold is chosen by visually inspecting the maps and taking into account across hemispheres symmetry and spatial homogeneity. The right histogram illustrates the improvement between two to three fibre populations exclusively for voxels that pass the criteria for one versus two models. This histogram is thresholded to indicate which voxels have two fixels and which have three fixels. The criteria for threshold is once more spatial homogeneity and cross-hemisphere symmetry. Maps depict a slicing through the template, with each voxel coloured according to the number of fixels it contains (yellow for one, blue for two, and orange for three).

### 4.3 Fixel-based GLM

To illustrate how the fixel-based framework can be put to use for statistical analysis, we conducted a GLM analysis to assess the changes in white matter fibre populations through ageing. For each subject, the HCP white matter fixel template was used as a prior to estimate fixel strength and orientation within each voxel.

**Fig. 10.**
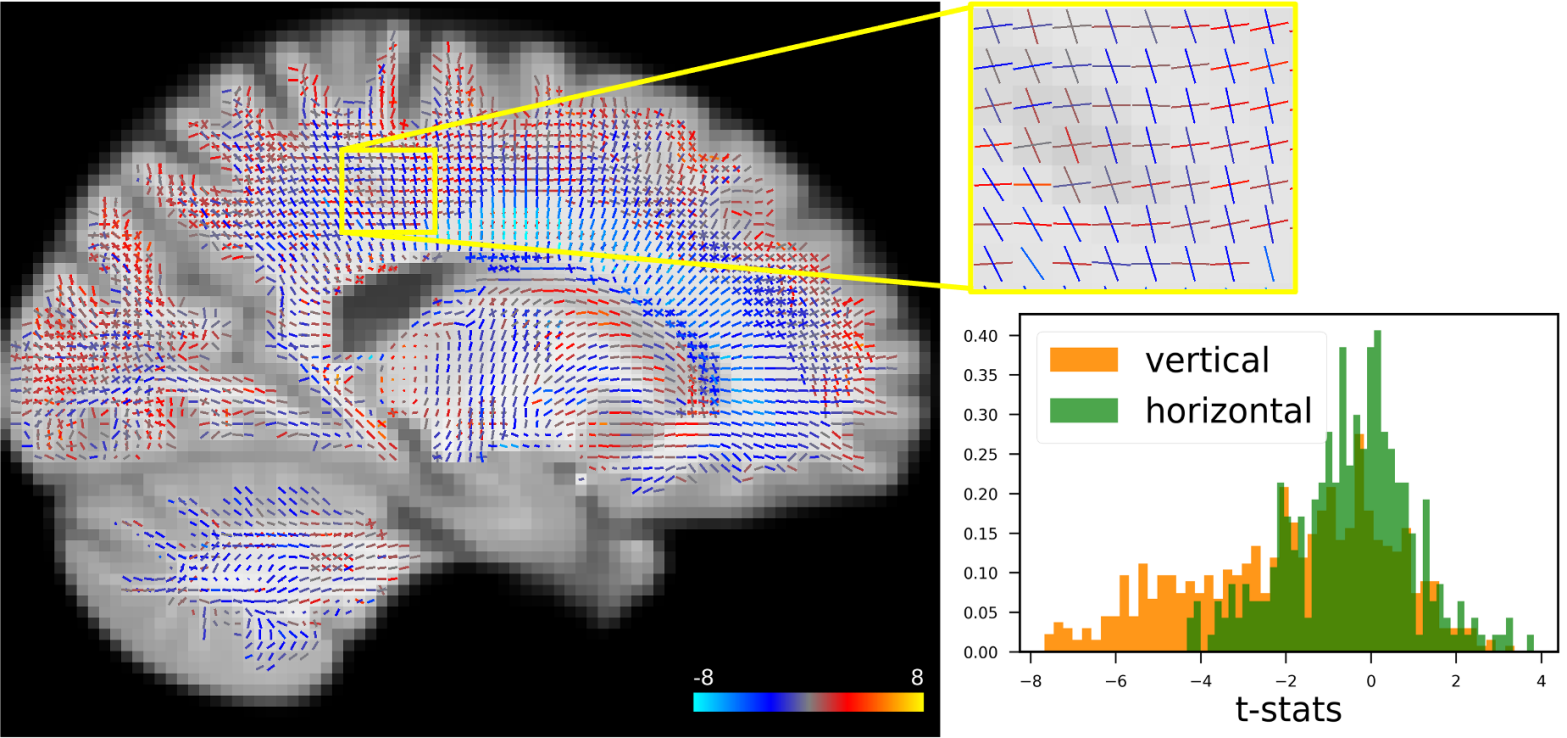
Left: A sample slice of t-statistics for fixels. Colour of lines indicate correlation with age (blue for negative, red for positive correlations). Right: Histogram of the t-statistics for horizontal and vertical fixels in the magnified region and five slices parallel to it. In this illustration, the distribution for vertical fibre populations is shown to be highly biased towards negative values, whereas the horizontal fibre distribution is centred around zero. In other words, on average, vertical (projection and commissural) tracts in this region degrade with age, while horizontal (association) tracts do not alter consistently.

Figure 10depicts a section of white matter with fixels coloured according to the t-statistics associated with the age regressor from the GLM: with red denoting an increase in fibre population signal fraction and blue denoting a reduction. To facilitate a better visual evaluation, a patch of crossing fibre areas is magnified. We observe both positive and negative changes in the region, with the majority of horizontal (anterior-posterior) fibres displaying positive changes and the majority of vertical (superior-inferior) fibres displaying negative changes. The histogram depicts the distribution of t-statistics separated for horizontal and vertical fibres inside the patch. The t-statistics for horizontal fibres are centred around zero, whereas the vertical population is skewed to the left.

**Fig. 11.**
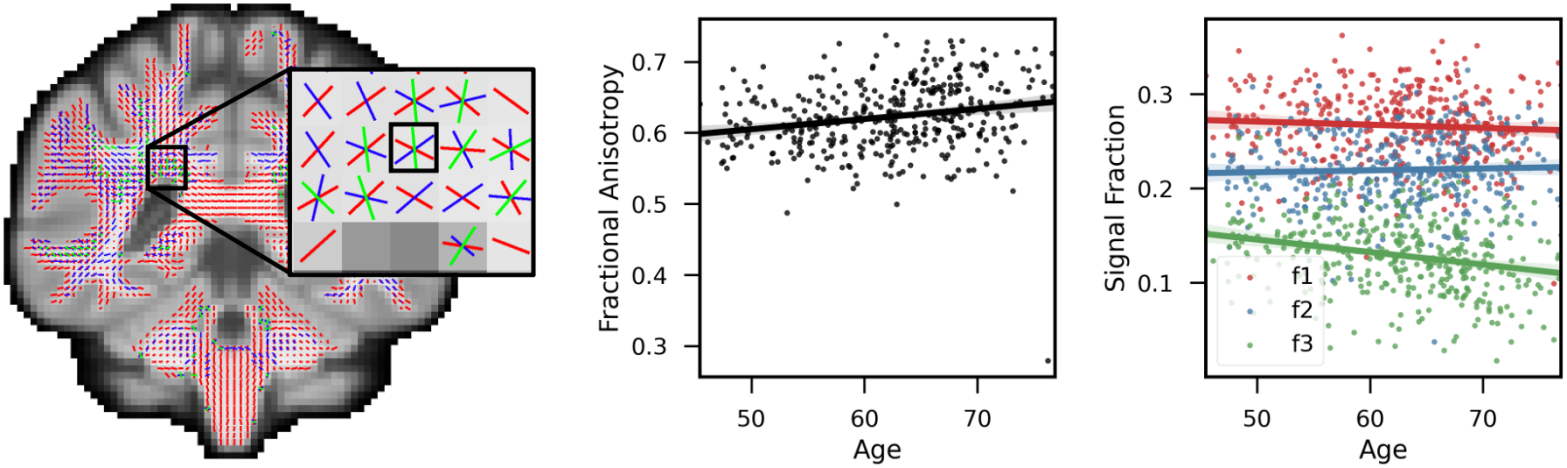
Comparison of FA and signal fractions in a sample voxel. Fractional anisotropy versus age for 400 subjects is shown in the scatter plot on the left (each dot represents a subject). In this voxel, the trend line (thick red line) suggests a positive FA–age correlation, which is conventionally attributed to an increase in fibre strength. The scatter plot on the right shows the signal proportions with trend lines for the three fibre populations present in this voxel (red for first, blue for second, and green for third fibre population). While the first two fibre populations in this voxel changed very little with age, the third population decreased substantially with age. As a result, anisotropy increases because diffusion decreases along the direction of the changed fibre population relative to the other two.

Figure 11displays the changes in signal fractions and fractional anisotropy for a representative deep white matter voxel as a function of age. The FA scatter plot trend indicates a rise in FA, which is sometimes interpreted as an increase in fibre integrity, when we would usually expect a decrease with ageing. Examining the fixel signal fractions helps us interpret this counter-intuitive result. The signal fractions for the three fibre populations in this voxel as a function of age are displayed in the scatter plot on the right. Rather than an increase in overall strength, the population of fibres with the lowest signal fraction is decreasing at a faster rate compared with the other two. This can explain the apparent increase in FA since anisotropy should increase, particularly when there is a large angle between this fibre population and the other two (Douaud et al., 2011) (also seeFig. 1).

**Fig. 12.**
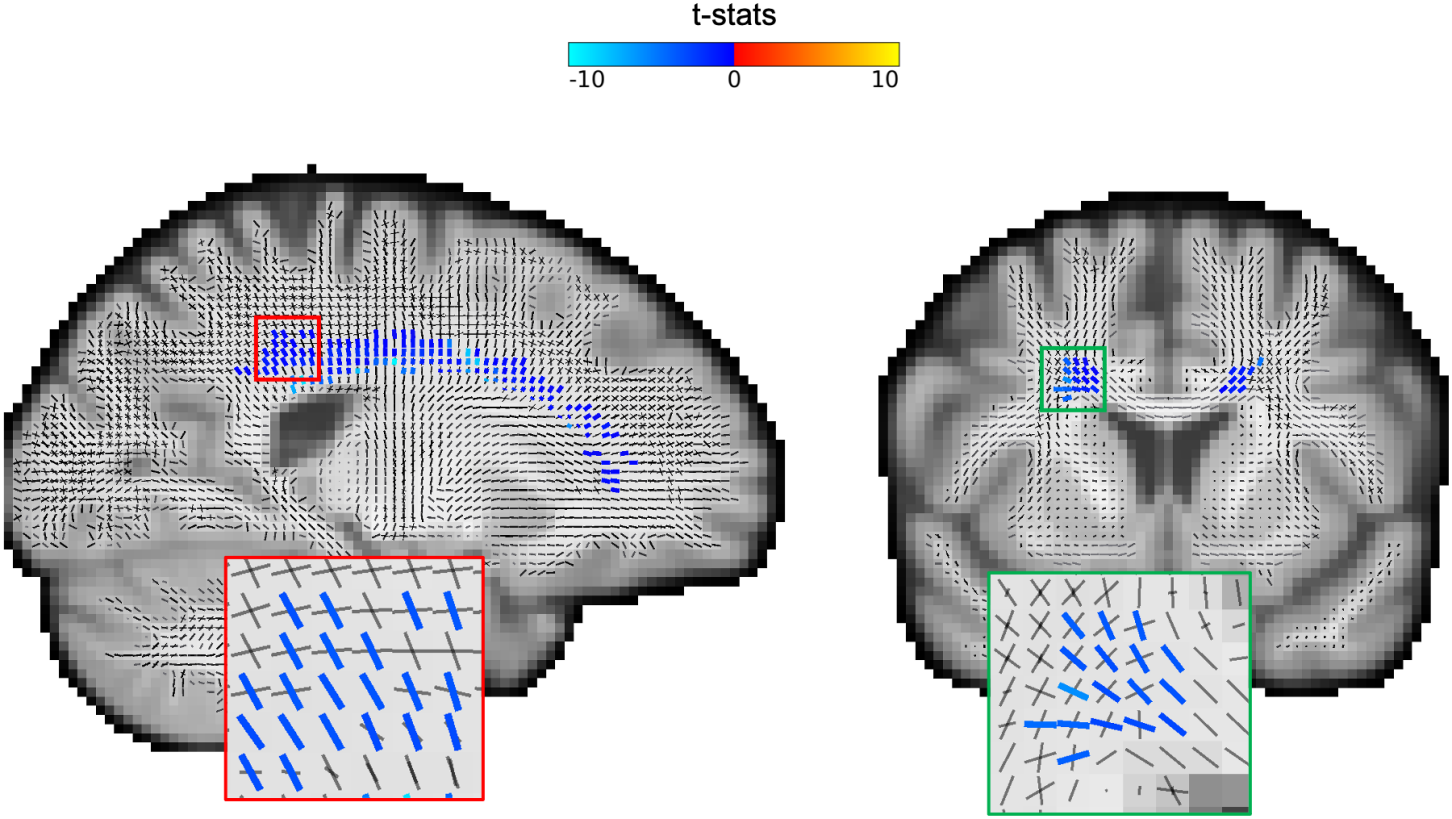
Statistically significant changes. Fixel-specific threshold-free cluster enhancement is applied to the t-statistics derived from the GLM analysis on fixel signal fractions versus age (E = 1, H = 3). Permutation tests with 5000 repetitions on the TFCE values are used to estimate p-values for each fixel. The maps depict the significant fixels (colour-coded based on the t-statistics) with corrected p < 0.05. Only negative changes in signal fractions pass the statistical significance test, and they are all situated in the same tract (body of corpus callosum) in both hemispheres.

Figure 12depicts a map of fixels with significant changes (p < 0.05) following multiple comparison correction with fixel-based threshold-free cluster enhancement. Only negative changes in the vicinity of the corpus callosum survived the significance test.

InFigure 13, we examine the changes with age in pairs of fixels forming crossing fibres in all voxels of the white matter. The figure displays the t-statistics of the first (x-axis) and second (y-axis) population of crossing fibres. Changes that occur in both populations of fibres and in the same direction (around the identity line in the scatter plots) represent coherent fixel changes, that is, voxel-wise change, as opposed to changes that occur in other directions (away from the identity line) that are fixel-specific changes. Significantly positive coherent changes (t-statistics in both fibre populations being more than 3) are shown by green dots in the scatter plot and displayed in the same colour on the maps. Voxels that exhibit substantial fixel-specific changes (t-statistics difference is greater than 3 in either of the fibre populations) are identified by red and displayed as such on the maps. We find that most coherent positive changes occur along the grey matter boundaries, suggesting changes in grey/white partial volume with age, whereas most fixel-specific changes occur in the deep white matter and likely capture fibre-specific changes in white matter organisation.

**Fig. 13.**
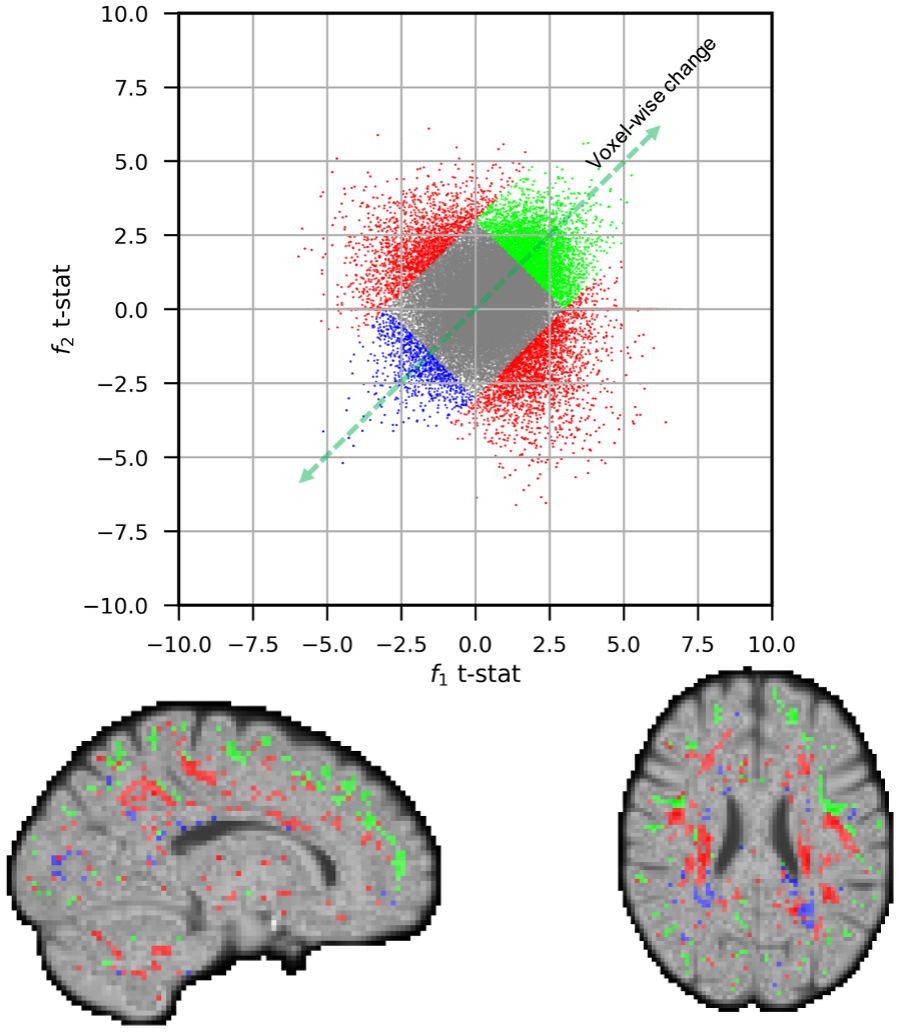
The scatter plot illustrates t-statistics for the relationship between signal fraction and age for the first and second fibre populations across all white matter voxels. Each data point corresponds to a single voxel and axes represent the first and second fixel’s t-statistics. In certain cases, both fixels within a voxel exhibit the same changes, resulting in points clustered near the identity line (voxel-wise changes). Points with significant changes of these instances (sum of t-values in both fixels being greater than 3 or lower than -3) are denoted in blue for negative changes and green for positive changes. Conversely, there are voxels where the fixel strengths change in different directions versus age, shown by data points deviating from the identity line. Voxels with considerable changes of this nature (difference in t-values being greater than 3) are highlighted in red. The accompanying maps reflect these results using the same colour scheme as the scatter plot. Notably, the majority of coherent positive changes at the voxel level are concentrated near the grey matter boundaries, while specific fixel changes or coherent negative changes are primarily observed in deep white matter regions. However, it is important to acknowledge the complexity associated with interpreting these changes. This complexity arises particularly from the constraint of the ball-and-stick model, wherein the sum of all fixel signal fractions is constant. Consequently, any alteration in the signal fraction of one stick must be offset by an opposite change in the signal fractions of the other sticks or the signal fraction of free water.

## 5 Discussion

In this study, we introduce a new approach for extracting fibre-specific measurements (fixels-based metrics) from diffusion MRI data. We used a hierarchical framework to fit the ball-and-sticks model to data from multiple subjects that offers some advantages over an individual fitting approach. First, the cross-subject fibre labelling issue is inherently resolved by the hierarchical structure of the model. This eliminates the need for post-hoc fibre matching, allowing us to directly compare fibre-specific parameters across individuals. The hierarchical structure also helps with robust parameter estimation for noisy subjects as it makes use of pooled data from multiple subjects. Natural expansions are viable in light of the hierarchical structure, such as hierarchically modelling subpopulations of individuals or employing a high-level model (such as age variables) as part of the inference process.

The hierarchical structure has some disadvantages as well. First and foremost, it is computationally more expensive than fitting the model to individuals separately. We addressed this issue by employing an EM algorithm, which converges within a few iterations and allows for both memory efficiency and convenient parallelisation across subjects. However, we note that once a template has been created, it can be utilised as a prior for fitting new subjects and would have the same computational costs as the individual fitting approach. Individual variations in the number of fixels within a voxel constitute another issue. Our method assumes that all subjects have the same number of fixels and is inflexible in this regard, but at least the hierarchical model enforces comparison of fixels with similar characteristics. Moreover, the hierarchical model requires accurate registration of white matter in terms of both location and local fibre orientation.

Also, it is essential to note that the fixel template is designed for application to relatively normal brain tissue. That is to say, the template’s use on severely damaged tissue, where fibre populations have been significantly displaced or destroyed due to a tumour or injury, may produce unreliable results. In other words, we advise against utilising the template for individuals with severe neurodegenerative conditions or extensive brain damage. However, one can create a template, by possibly using a more suited microstructure model, from a cohort with specific tissue damage and then apply it to similar datasets.

While the proposed framework is one option to extract fibre-specific measurements, it is not the only one; the MRTRIX software (Tournier et al., 2019) also has a very solid pipeline implemented that has been used in many studies. There are two key differences compared with our approach. Firstly, we estimate individual fibre orientations using the ball-and-sticks model while the other approach uses spherical deconvolution. Secondly, they solve the fibre assignment across subjects post-hoc, after model fitting, whereas we perform it as part of the modelling process. Both methods provide proxy measures for fibre strength. Please seeAppendix Ifor a comparison of the results from the analysis presented in this paper.

As of now, the diffusion tensor metrics such as FA and MD are the most frequently used measurements to examine the integrity of brain tissue. A GLM analysis for FA vs age reveals that a number of brain areas exhibit significant positive correlation with age. While a decrease of FA is often interpreted as reduced tract integrity, increases in FA are more difficult to interpret. InFigure 11, we provided a concrete example of real data demonstrating that a drop in the signal fraction of a crossing fibre in an example voxel in deep white matter is contributing to a rise in FA. Also, after applying the cluster corrections, the results of the fixel-based GLM analysis show that all significant changes in the fibre strengths are negative. This would imply that the FA increases are confounded by the geometrical structure of the tracts and their relative alterations within each voxel.

### 5.1 Fixel template creation and usage

We have applied the proposed methods on a few subjects from the HCP young adult dataset to produce a white matter fixel template. This template contains the probability distribution for the orientation of fibre populations present in each white matter voxel in the Oxford multimodal template (OMM-1), which can be used in the future for fibre-specific statistical analysis. It may be essential to create a new template if a cohort-specific template is required (e.g., for disease or a different age range), if using a different base diffusion model (other than ball-and-sticks), or if using a structural template other than OMM-1.

It is worth re-emphasising that while the hierarchical model can be fitted to a cohort of subjects using our EM algorithm, the resulting group parameters constitute a template that can be employed in its own right as a prior to fit additional subjects that are not part of the same cohort. This is not only useful for computational reasons. It can also benefit in fitting lower quality data than those used to create the template. For instance, it is well established that in diffusion modelling, there is a tension between acquiring multiple shells to have a better estimate of the diffusion parameters (e.g., ADC and kurtosis, etc.) and acquiring multiple directions to have more accurate crossing fibre modelling. Most datasets compromise on one or the other. The template can be created using high-quality, multi-shell, and multi-directions data, such as the HCP data, and then used as a prior to constrain the analysis of lower quality data. We have used exactly this approach here, with a template created using HCP dataset, and deployed to analyse the UK Biobank data. Nevertheless, when adapting the template to datasets obtained through different protocols, it is advisable to exclude the priors on diffusivities. This is because these measurements might be influenced by the specifics of the acquisition protocols and the strength of the gradients used. Also, it is essential to consider that while the priors pertain to orientations and not the signal fractions, these two may remain interconnected. In cases where the reconstruction of an existing fixel within a voxel is unsuccessful, it is plausible that this failure could introduce a bias in the signal fraction estimation of other fixels within the same voxel.

In this study, the hierarchical structure was applied to all parameters of the ball-and-sticks model, except for the baseline signal (S0) parameter. This exclusion was made to allow for higher intra-subject variability in S0 as a result of potential artefacts such as bias field. Essentially, only stick orientation parameters must be maintained within the hierarchy to ensure consistent labelling of fibres between subjects, the rest of the parameters can be removed from the hierarchical structure. Incorporating a parameter into the hierarchical model can help with regularising its estimates, that is particularly useful in subjects with noisy data. However, it may also introduce biases towards the global mean; which in turn can bias the estimates towards the majority group or reduce the statistical power. Therefore, it is advisable to exclude microstructural parameters intended for comparison across groups from the hierarchical structure. Also, we do not recommend using the stick orientation parameters for statistical analysis as they might be slightly biased due to the hierarchical prior.

In order to determine how many fixels are present in each voxel, we have relied on the increase in log-likelihood as a measure of goodness of fit. In determining an appropriate cut-off for this metric, we take into account the spatial coherence in the produced maps and whether they are consistent with the information we currently have on the total number of tracts in each region. To determine the thresholds, one may also use metrics such as AIC and BIC. However, there is always an arbitrary trade-off between the number of free parameters in the model and its goodness of fit. In any case, the thresholds are fairly flexible and can be re-adjusted with minimal effort if there is a compelling reason to do so. Additionally, automated methods for determining the number of fibre populations may be developed and readily integrated into this framework in the future.

In addition to the free parameters that are fitted using data, the hierarchical model also includes user-set hyperparameters$\left(\alpha_{g},\alpha_{n}\right)$that constrain the prior on the group variance and the noise variance. Having a high$\alpha_{g}$imposes a greater penalty on group variances to approach zero, hence increasing the chance to favour individual differences. A high$\alpha_{n}$increases the penalty for estimating high noise variance. Changing either of these parameters within a suitable range, however, should not significantly alter the model’s behaviour.

### 5.2 Future directions

The hierarchical structure can be enhanced further in a number of ways. The template can be created in a nested hierarchical structure for multiple groups of subjects, and comparisons can then be made between group parameters. Another modification is to employ a regression model to incorporate independent variables (such as age) into the hierarchical structure. Moreover, in order to better handle noisy subjects, one can additionally include the uncertainty in subject parameter estimates into the model and convert it to a mixed-effect analysis.

The fixel template is created by employing the ball-and-sticks model to extract the orientations of the fibre populations within each voxel. The ball-and-stick model was chosen as it is a well-tested model designed to robustly extract crossing fibres in the brains white matter. However, this does not imply that the tract-specific metrics are restricted to these model parameters. The presented template can be used as a starting point for developing more complex and specific biophysical models to fit the data. For instance, the template can be used to fit fibre dispersion or multi-compartment models (e.g., NODDI (H. Zhang et al., 2012)) with crossing fibres by utilising the information on the number and orientation of fibre populations in each voxel. The parameters of those biophysical models can subsequently be employed as fixel-specific measurements that are compared across subjects.

As an illustration of the proposed method, we have employed a GLM framework to conduct fixel-specific statistical analysis in this study. The steps for performing such an analysis and the results to anticipate are laid out in detail here. Age has been used as an independent variable to clarify usage, but we hope that the tool will be useful to explore fibre-specific alterations as a function of disease or other biological processes. Moreover, it is possible to explore fibre-specific changes in the brain by integrating these measurements with data-driven techniques such as Independent Component Analysis (ICA) or Canonical Correlation Analysis (CCA). To achieve this, one can create a fixel-by-subject matrix by concatenating all the fixels’ data, and then apply matrix factorisation algorithms to estimate patterns of variation within the fixels. This approach helps identify modes of variation in the population, providing insights into how fixel strength varies across subjects. Additionally, the loadings obtained from this analysis can be valuable for identifying potentially interesting and meaningful individual differences. Moreover, tractography approaches can be applied to the template to reconstruct the major white matter tracts, enabling the across-subjects comparison of brain structure along the tracts.

In order to increase the power of statistical tests for fixel-based data, here we present a modified version of threshold-free cluster enhancement. Similar to the original TFCE technique, this method utilises two hyperparameters (E and H) to adjust how heavily cluster extent and height play towards promoting significant clusters. In this study, we have used the original method’s (E=3, H=2) values for these parameters, which have been shown through simulations to be appropriate for voxel-wise data. Future development may involve adjusting them to accommodate for the different neighbourhood structure of fixel-based data compared with that of voxel-based data.

## Figures and Tables

**Fig. 14.**
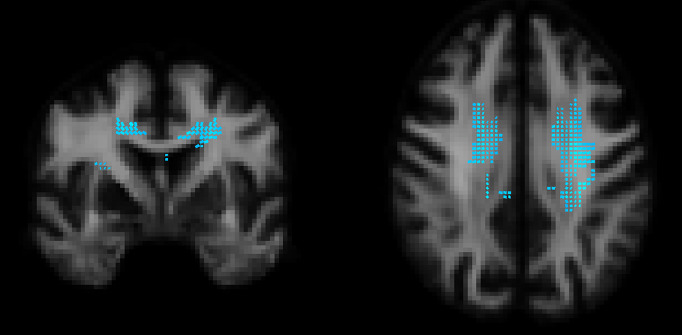

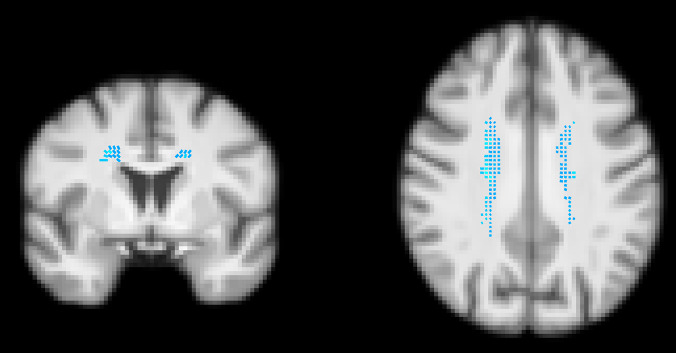
Illustration of the outcomes of the GLM analysis obtained from the MRtrix FBA pipeline (top) and utilising WHIM (bottom). In the top plot, the fixels displaying a p-value below 0.05 after applying cluster enhancement are superimposed on the zeroth order template FOD coefficient. The results from our pipeline, derived from the same data but using WHIM pipeline, are presented at the bottom overlaid on the OMM1 template.

## Data Availability

WHIM (White matter HIerarchical Modelling) is an open-source software package and freely accessible fromhttps://git.fmrib.ox.ac.uk/hossein/whim. WHIM is versatile and allows for the execution of full hierarchical inference, the generation of new templates, and the analysis of new data using available templates. WHIM will be integrated into new versions of FSL. This study utilised data from the UK Biobank and the Human Connectome Project, which are accessible in accordance with the providers’ policies.

## Appendix I

### Comparison with MRtrix

To provide users with a comparative overview of our proposed approach in relation to the state-of-the-art method (MRtrix), we conducted a parallel analysis and present the statistical outcomes. For MRtrix, we followed the pipeline detailed in the manual available at MRtrix documentation website. The identical dataset and design matrix employed for our approach were used (refer toSection 3for further details).

Figure 14illustrates the significant fixels with a corrected p-value less than 0.05 in two selected sample slices. The results exhibit a qualitative similarity, indicating a reduction in apparent fibre density (equivalent to fibre signal fraction in our approach) within the same regions. However, it is important to note that direct quantification of the similarities and differences in the results is not straightforward due to the distinct template spaces utilised. The WHIM fixel template is situated in the structural template space (OMM1), while the MRtrix fixel template is established using Fibre Orientation Distributions (FODs).

## References

[b1] Alexander , D. , Pierpaoli , C. , Basser , P. , & Gee , J. ( 2001 ). Spatial transformations of diffusion tensor magnetic resonance images . IEEE Transactions on Medical Imaging , 20 ( 11 ), 1131 – 1139 . 10.1109/42.963816 11700739

[b2] Alfaro-Almagro , F. , Jenkinson , M. , Bangerter , N. K. , Andersson , J. L. , Griffanti , L. , Douaud , G. , Sotiropoulos , S. N. , Jbabdi , S. , Hernandez-Fernandez , M. , Vallee , E. , Vidaurre , D. , Webster , M. , McCarthy , P. , Rorden , C. , Daducci , A. , Alexander , D. C. , Zhang , H. , Dragonu , I. , Matthews , P. M. , … Smith , S. M. ( 2018 ). Image processing and quality control for the first 10,000 brain imaging datasets from UK Biobank . NeuroImage , 166 , 400 – 424 . 10.1016/j.neuroimage.2017.10.034 29079522 PMC5770339

[b3] Arthofer , C. , Smith , S. , Jenkinson , M. , Andersson , J. , & Lange , F. ( 2021 ). Multimodal MRI template construction from UK Biobank: Oxford-MM-0 . Organisation for Human Brain Mapping (OHBM) . 10.1101/2023.11.30.569378

[b4] Ashburner , J. , & Friston , K. J. ( 2000 ). Voxel-based morphometry—The methods . NeuroImage , 11 ( 6 ), 805 – 821 . 10.1006/nimg.2000.0582 10860804

[b5] Behrens , T. , Berg , H. J. , Jbabdi , S. , Rushworth , M. , & Woolrich , M. ( 2007 ). Probabilistic diffusion tractography with multiple fibre orientations: What can we gain? NeuroImage , 34 ( 1 ), 144 – 155 . 10.1016/j.neuroimage.2006.09.018 17070705 PMC7116582

[b6] Dhollander , T. , Clemente , A. , Singh , M. , Boonstra , F. , Civier , O. , Duque , J. D. , Egorova , N. , Enticott , P. , Fuelscher , I. , Gajamange , S. , Genc , S. , Gottlieb , E. , Hyde , C. , Imms , P. , Kelly , C. , Kirkovski , M. , Kolbe , S. , Liang , X. , Malhotra , A. , … Caeyenberghs , K. ( 2021 ). Fixel-based analysis of diffusion MRI: Methods, applications, challenges and opportunities . NeuroImage , 241 , 118417 . 10.1016/j.neuroimage.2021.118417 34298083

[b7] Douaud , G. , Jbabdi , S. , Behrens , T. E. , Menke , R. A. , Gass , A. , Monsch , A. U. , Rao , A. , Whitcher , B. , Kindlmann , G. , Matthews , P. M. , & Smith , S. ( 2011 ). DTI measures in crossing-fibre areas: Increased diffusion anisotropy reveals early white matter alteration in MCI and mild Alzheimer’s disease . NeuroImage , 55 ( 3 ), 880 – 890 . 10.1016/j.neuroimage.2010.12.008 21182970 PMC7116583

[b8] Hong , X. , Arlinghaus , L. R. , & Anderson , A. W. ( 2009 ). Spatial normalization of the fiber orientation distribution based on high angular resolution diffusion imaging data . Magnetic Resonance in Medicine , 61 ( 6 ), 1520 – 1527 . 10.1002/mrm.21916 19353649 PMC2774933

[b9] Jbabdi , S. , Behrens , T. E. , & Smith , S. M. ( 2010 ). Crossing fibres in tract-based spatial statistics . NeuroImage , 49 ( 1 ), 249 – 256 . 10.1016/j.neuroimage.2009.08.039 19712743

[b10] Jenkinson , M. , Beckmann , C. F. , Behrens , T. E. , Woolrich , M. W. , & Smith , S. M. ( 2012 ). FSL . NeuroImage , 62 ( 2 ), 782 – 790 . 10.1016/j.neuroimage.2011.09.015 21979382

[b11] Johansen-Berg , H. , & Behrens , T. E. J. ( 2014 ). Diffusion MRI: From quantitative measurement to in-vivo neuroanatomy ( 2nd ed.) [OCLC: 899573164]. Elsevier Science . 10.1016/b978-0-12-374709-9.00023-7

[b12] Jones , D. K. (Ed.). ( 2010 ). Diffusion MRI: Theory, methods, and application . Oxford University Press . 10.1093/med/9780195369779.003.0015

[b13] Lange , F. J. , Ashburner , J. , Smith , S. M. , & Andersson , J. L. ( 2020 ). A symmetric prior for the regularisation of elastic deformations: Improved anatomical plausibility in nonlinear image registration . NeuroImage , 219 , 116962 . 10.1016/j.neuroimage.2020.116962 32497785 PMC7610794

[b14] Miller , K. L. , Alfaro-Almagro , F. , Bangerter , N. K. , Thomas , D. L. , Yacoub , E. , Xu , J. , Bartsch , A. J. , Jbabdi , S. , Sotiropoulos , S. N. , Andersson , J. L. R. , Griffanti , L. , Douaud , G. e. , Okell , T. W. , Weale , P. , Dragonu , I. , Garratt , S. , Hudson , S. , Collins , R. , Jenkinson , M. , … Smith , S. M. ( 2016 ). Multimodal population brain imaging in the UK Biobank prospective epidemiological study . Nature Neuroscience , 19 ( 11 ), 1523 – 1536 . 10.1038/nn.4393 27643430 PMC5086094

[b15] Nelder , J. A. , & Mead , R. ( 1965 ). A simplex method for function minimization . The Computer Journal , 7 ( 4 ), 308 – 313 . 10.1093/comjnl/7.4.308

[b16] Raffelt , D. , Tournier , J.-D. , Crozier , S. , Connelly , A. , & Salvado , O. ( 2012 ). Reorientation of fiber orientation distributions using apodized point spread functions . Magnetic Resonance in Medicine , 67 ( 3 ), 844 – 855 . 10.1002/mrm.23058 22183751

[b17] Raffelt , D. , Tournier , J.-D. , Fripp , J. , Crozier , S. , Connelly , A. , & Salvado , O. ( 2011 ). Symmetric diffeomorphic registration of fibre orientation distributions . NeuroImage , 56 ( 3 ), 1171 – 1180 . 10.1016/j.neuroimage.2011.02.014 21316463

[b18] Raffelt , D. A. , Tournier , J.-D. , Smith , R. E. , Vaughan , D. N. , Jackson , G. , Ridgway , G. R. , & Connelly , A. ( 2017 ). Investigating white matter fibre density and morphology using fixel-based analysis . NeuroImage , 144 ( 0 ), 58 – 73 . 10.1016/j.neuroimage.2016.09.029 27639350 PMC5182031

[b19] Scholz , J. , Klein , M. C. , Behrens , T. E. J. , & Johansen-Berg , H. ( 2009 ). Training induces changes in white-matter architecture . Nature of Neuroscience , 12 ( 11 ), 1370 – 1371 . 10.1038/nn.2412 19820707 PMC2770457

[b20] Smith , S. , & Nichols , T. ( 2009 ). Threshold-free cluster enhancement: Addressing problems of smoothing, threshold dependence and localisation in cluster inference . NeuroImage , 44 ( 1 ), 83 – 98 . 10.1016/j.neuroimage.2008.03.061 18501637

[b21] Smith , S. M. , Jenkinson , M. , Johansen-Berg , H. , Rueckert , D. , Nichols , T. E. , Mackay , C. E. , Watkins , K. E. , Ciccarelli , O. , Cader , M. Z. , Matthews , P. M. , & Behrens , T. E. ( 2006 ). Tract-based spatial statistics: Voxelwise analysis of multi-subject diffusion data . NeuroImage , 31 ( 4 ), 1487 – 1505 . 10.1016/j.neuroimage.2006.02.024 16624579

[b22] Sotiropoulos , S. N. , Jbabdi , S. , Xu , J. , Andersson , J. L. , Moeller , S. , Auerbach , E. J. , Glasser , M. F. , Hernandez , M. , Sapiro , G. , Jenkinson , M. , Feinberg , D. A. , Yacoub , E. , Lenglet , C. , Van Essen , D. C. , Ugurbil , K. , & Behrens , T. E. J. ( 2013 ). Advances in diffusion MRI acquisition and processing in the Human Connectome Project . NeuroImage , 80 , 125 – 143 . 10.1016/j.neuroimage.2013.05.057 23702418 PMC3720790

[b23] Tournier , J.-D. , Smith , R. , Raffelt , D. , Tabbara , R. , Dhollander , T. , Pietsch , M. , Christiaens , D. , Jeurissen , B. , Yeh , C.-H. , & Connelly , A. ( 2019 ). MRtrix3: A fast, flexible and open software framework for medical image processing and visualisation (tech. rep.). Cold Spring Harbor Laboratory . 10.1016/j.neuroimage.2019.116137 31473352

[b24] Van Essen , D. C. , Smith , S. M. , Barch , D. M. , Behrens , T. E. , Yacoub , E. , & Ugurbil , K. ( 2013 ). The WU-Minn Human Connectome Project: An overview . NeuroImage , 80 , 62 – 79 . 10.1016/j.neuroimage.2013.05.041 23684880 PMC3724347

[b25] Virtanen , P. , Gommers , R. , Oliphant , T. E. , Haberland , M. , Reddy , T. , Cournapeau , D. , Burovski , E. , Peterson , P. , Weckesser , W. , Bright , J. , van der Walt , J. , Brett , M. , Wilson , J. , Millman , K. J. , Mayorov , N. , Nelson , A. R. J. , Jones , E. , Kern , R. , Larson , E. , … SciPy 1.0 Contributors . ( 2020 ). SciPy 1.0: Fundamental algorithms for scientific computing in Python . Nature Methods , 17 , 261 – 272 . 10.1038/s41592-019-0686-2 32015543 PMC7056644

[b26] Zhang , H. , Schneider , T. , Wheeler-Kingshott , C. A. , & Alexander , D. C. ( 2012 ). NODDI: Practical in vivo neurite orientation dispersion and density imaging of the human brain . NeuroImage , 61 ( 4 ), 1000 – 1016 . 10.1016/j.neuroimage.2012.03.072 22484410

[b27] Zhang , H. , Yushkevich , P. A. , Alexander , D. C. , & Gee , J. C. ( 2006 ). Deformable registration of diffusion tensor mr images with explicit orientation optimization [The Eighth International Conference on Medical Imaging and Computer Assisted Intervention – MICCAI 2005] . Medical Image Analysis , 10 ( 5 ), 764 – 785 . 10.1016/j.media.2006.06.004 16899392

[b28] Zhang , P. , Niethammer , M. , Shen , D. , & Yap , P.-T. ( 2014 ). Large deformation diffeomorphic registration of diffusion-weighted imaging data [Special Issue on the 2013 Conference on Medical Image Computing and Computer Assisted Intervention] . Medical Image Analysis , 18 ( 8 ), 1290 – 1298 . 10.1016/j.media.2014.06.012 25106710 PMC4213863

[b29] Zhang , Y. , Brady , M. , & Smith , S. ( 2001 ). Segmentation of brain MR images through a hidden Markov random field model and the expectation-maximization algorithm . IEEE Transactions on Medical Imaging , 20 ( 1 ), 45 – 57 . 10.1109/42.906424 11293691

